# Mortality Assessment in Patients with Cardiovascular Disease and COVID-19: A Systematic Review and Meta-Analysis

**DOI:** 10.3390/ijms27104375

**Published:** 2026-05-14

**Authors:** Małgorzata Jarończyk, Jarosław Walory

**Affiliations:** 1 Paediatric Regional Centre for Digital Medicine, The Children’s Memorial Health Institute, 04-730 Warsaw, Poland; 2 Department of Medical Biology, Digital Medicine Center–Biobank, Cardinal Stefan Wyszynski National Institute of Cardiology, 04-628 Warsaw, Poland; jwalory@gmail.com

**Keywords:** cardiovascular disease, chronic kidney disease, COVID-19, diabetes mellitus, hypertension, mortality

## Abstract

The COVID-19 pandemic has had a profound impact on global health, especially among patients with cardiovascular disease (CVD) and the existence of additional conditions such as diabetes (DM), hypertension (HT), and chronic kidney disease (CKD) can have a significant impact on survival rates. The aim of this study was to determine the mortality rate in patients with CVD and the impact of other comorbidities on the death of patients with COVID-19. This systematic review was conducted using PubMed, EMBASE, and Google Scholar databases from August 2020 to June 2025. Inclusion criteria were patients with cardiovascular disease and associated comorbidities during the COVID-19 pandemic. Article selection was limited to articles published in English and Polish. Statistical analysis using a random-effects model was performed using STATA software. Heterogeneity between studies was examined, and a funnel plot for publication bias was generated. The higher mortality rates (OR = 3.00, 95% CI: 2.06–4.38) for patients with cardiovascular disease were observed. In the group of patients with comorbidities such as hypertension and diabetes mellitus the risk of death was also determined and for HT was OR = 1.94, 95% CI, 1.50–2.52 and for DM OR = 2.17, 95% CI: 1.64–2.86. The mortality in the chronic kidney disease group was higher than for HT and DM (OR = 3.91, 95% CI: 2.50–6.10). The risk of death is three times higher for patients with COVID-19 and CVD. High mortality risk is also linked to diabetes and hypertension but for chronic kidney disease patients increased up to four times.

## 1. Introduction

The COVID-19 pandemic has been a major challenge for all healthcare systems [1]. In the period from 1 January 2020 to 31 December 2021, the countries with the highest excess mortality rates were Poland, India, the Russian Federation, Indonesia and the USA [2]. Many studies have been published on various aspects of COVID-19 [3,4,5]. Cardiovascular disease (CVD) and COVID-19 have become a major area of public health concern, especially in the context of the impact of the pandemic on people with pre-existing comorbidities. Various studies have shown that patients with circulatory system diseases have a significantly higher mortality rate after Severe Acute Respiratory Syndrome Coronavirus 2 (SARS-CoV-2) infection, and comorbidities significantly increase the severity of COVID-19 infection [6,7]. To date, few systematic reviews on mortality in the course of COVID-19 disease with concomitant cardiovascular diseases have been conducted worldwide, but they have not included reports of mortality from Poland.

In this review, we assess the impact of SARS-COV-2 infection and the impact of comorbidities on mortality in patients with CVD during the pandemic.

Understanding the impact of COVID-19 on people with CVD can help healthcare providers more effectively manage at-risk populations, ultimately reducing mortality. Although this study provides critical insights, it is important to acknowledge that the evolving nature of SARS-CoV-2 and its variants may impact the significance of these findings over time, requiring ongoing updates to research in this area.

Our meta-analysis included data from a geographically diverse population, with Poland contributing as one of the represented countries, allowing for assessment of geographic variation in mortality risk.

The main outcome studied was mortality in a group of patients infected with SARS-CoV-2 CVD Other comorbidities (hypertension (HT), diabetes mellitus (DM), and chronic kidney disease (CKD)) were analyzed as secondary outcomes to provide a broader clinical context and because they were frequently co-reported in the included studies.

## 2. Materials and Methods

**Figure 1:**
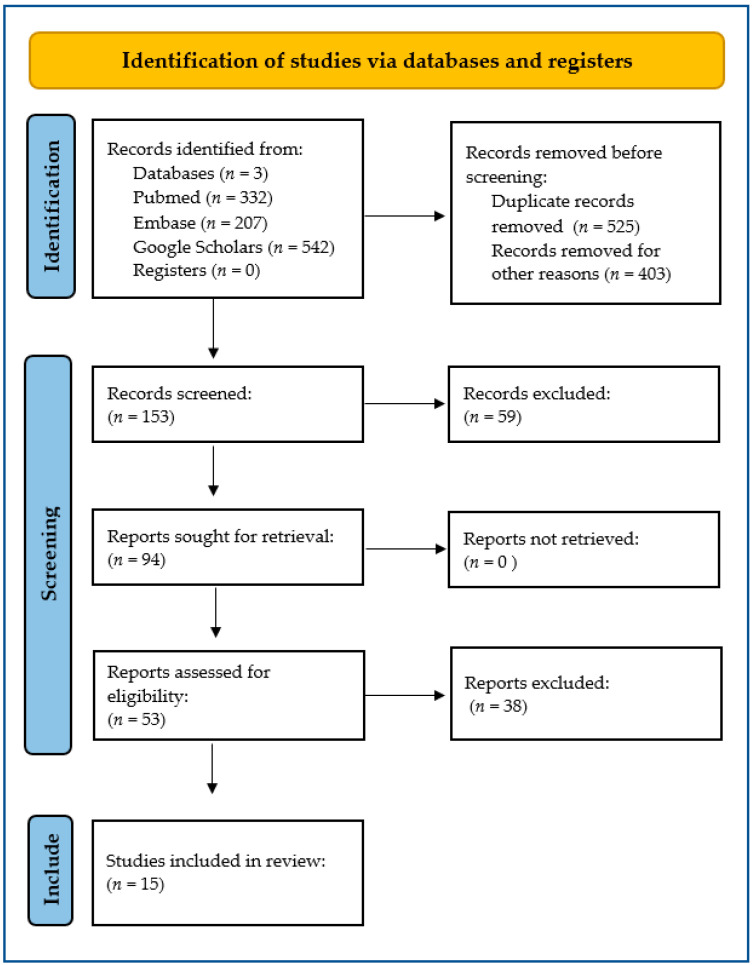
The flow diagram for the systematic review.

The Preferred Reporting Items for Systematic Reviews and Meta-Analyses (PRISMA) framework was used in this systematic review (Figure 1, Supplementary Table S1) [8]. 

The research question for this systematic review was developed using the PECO framework (Population, Exposure, Comparison, Outcome), with a particular focus on patients with cardiovascular disease infected by COVID-19. 

(P) The population included adults (≥18 years) with confirmed SARS-CoV-2 infection and CVD, hospitalized or non-hospitalized patients. The exclusion criterion was pediatric populations (<18 years).

(E) The exposure of interest was the presence of additional comorbidities—such as CKD, DM, HT—reflecting the burden of multimorbidity in this group. 

(C) A comparison was made between non-survivors (mortality) and survivors (recovery or discharge).

(O) The primary outcome was defined as all-cause mortality. Inclusion criteria were data containing or enabling extraction of effects (e.g., odds ratio (OR), risk ratio (RR), hazard ratio (HR)). Exclusion criteria were insufficient data for analysis.

This structured approach facilitated a comprehensive evaluation of how coexisting chronic conditions influence prognosis in COVID-19 patients with underlying cardiovascular disease.

A systematic search of medical databases was performed for publications from the period of August 2020–June 2025 using PubMed, EMBASE and Google Scholar. Studies that report SARS-CoV-2 infection in CVD patients, including retrospective cohort, observational cohort and prospective cohort studies, were selected for this systematic review. We excluded review articles, case reports, commentary and editorials and also animal studies. A panel of two reviewers did a blinded screening of titles, abstracts and full texts using the appropriate criteria and selected studies that were further included in the systematic review. The search keywords with the combinations used were “coronavirus”, “COVID-19”, “Mortality”, “Clinical outcomes”, “Clinical course”, and “Cardiovascular disease” using Boolean connectors “And”, “Or” and “Not”. 

Duplicate references were removed from the list of articles at the identification level (Figure 1). The list was limited to those published in English and Polish. In the first stage of screening, 59 studies were excluded due to lack of data on comorbidities. Thirty-eight studies were excluded after full-text review due to lack of significant results or inadequate study design to allow further analysis. Fifteen studies met all predefined eligibility criteria and were included in the final analysis.

Cardiovascular diseases included chronic coronary syndrome, coronary artery disease, coronary heart disease, peripheral vascular disease, stroke, ischemic cardiomyopathy, acute cardiac injury, chronic cardiovascular disease, and cardiac disease. In the case of the chronic kidney disease, we included dialysis and acute renal injury in one of the studies. In the case of hypertension we included studies with arterial hypertension, and in the case of diabetes in one of the studies we included diabetes with end organ damage.

The performed meta-analysis uses a random-effects model estimated using Bayes statistics to calculate pooled ORs and confidence intervals (CIs). STATA software version 18.5 was used [9]. The *I*^2^ statistics were used to evaluate the degree of heterogeneity. To determine the statistical significance of the effect of small studies (publication bias), funnel plots were assessed, and Egger’s regression asymmetry test was used (random-effects model, method: Empirical Bayes).

## 3. Results

Table 1 presents the characteristics of 68,963 patients from 15 cohort studies, while the distribution of participants across nine countries (Brazil, China, India, Italy, Poland, Russia, South Korea, the United Kingdom, and the USA) is summarized in Table 2. Males accounted for approximately 51% of the total population, and the median age exceeded 51 years.

The main outcome studied was mortality in a group of patients infected with SARS-CoV-2 with cardiovascular disease, and the second outcome was mortality among individuals burdened with comorbidities.

The proportions of patients with comorbidities in the study population were: 10.7% CVD (7413), 27.3% HT (18,812), 13.1% DM (9057) and 3.5% CKD (2418). The mortality rates of patients with comorbidities in the study population were: 23.3% (1726/7413), 16.3%, (3071/18,812) 18.3% (1661/9057), and 35.5% (858/2418) for CVD, HT, DM and CKD, respectively. The total mortality rate was 9.7% (6736/68,963).

The meta-analysis revealed significant heterogeneity in the studied variables: 93.66%, 84.89%, 88.91%, and 92.39% for CVD, HT, DM and CKD, respectively.

**Figure 2:**
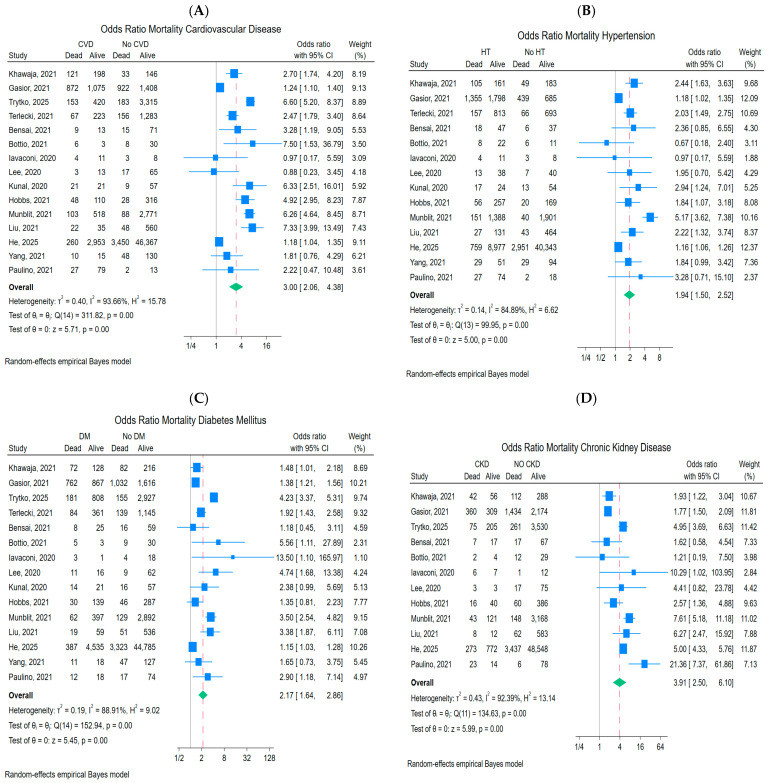
Forest plot of mortality rate for patients with vs. without: (**A**), cardiovascular disease; (**B**), hypertension; (**C**), diabetes mellitus; (**D**), chronic kidney disease showing the pooled odds ratio (OR) with 95% confidence intervals (95% CIs). The references to 15 cohort studies: Khawaja, 2021 [10]; Gąsior, 2021 [11]; Trytko, 2025 [12]; Terlecki 2021 [13]; Bensai, 2021 [14]; Bottio, 2021 [15]; Iavaconi, 2020 [16]; Lee, 2020 [17]; Kunal, 2020 [18]; Hobbs, 2021 [19]; Munblit, 2021 [20]; Liu, 2021 [21]; He, 2025 [22]; Yang, 2021 [23]; Paulino, 2021 [24].

**Figure 3:**
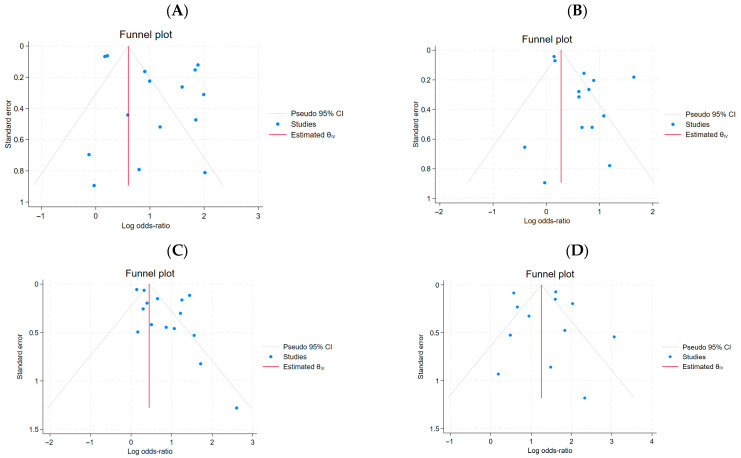
Funnel plots of publication bias analysis for studies included: (**A**), cardiovascular disease; (**B**), hypertension; (**C**), diabetes mellitus; (**D**), chronic kidney disease.

Pre-existing cardiovascular diseases were found to significantly increase the risk of mortality in COVID-19 patients. Those with cardiovascular conditions were nearly three times more likely to experience fatal outcomes. From the cohort group of 7413 (10.7%) individuals with cardiovascular disease, a total of 1726 (23.3%) died. The presence of cardiovascular disease was associated with a higher odds ratio of mortality (OR = 3.0, 95% CI: 2.06–4.38, *p* < 0.001; Figure 2A). Despite high heterogeneity (*I*^2^ = 93.66%), no systematic asymmetry related to study precision was observed. (OR = 3.0, 95% CI: 2.06–4.38, *p*< 0.001, Figure 2A). The funnel plot and Egger’s test (*β*_1_ = −0.13, SE *β*_1_ = 0.82, *z* = −0.16, *p* = 0.87) suggested no evidence of reporting bias for small-study effects (Figure 3A). 

In this systematic review we also analyzed the effect of comorbidities such as HT, DM and CKD on patient mortality. 

In the group of individuals with HT comorbidity, higher risk of death (OR = 1.94, 95% CI: 1.50–2.52) was observed in comparison to the control group and *p* < 0.001 (Figure 2B). Among diabetic patients, a higher mortality rate was also noted (OR = 2.17, 95% CI: 1.64–2.86) and was statistically significant *p* < 0.001 as shown in Figure 2C. Pooled analysis of 12 studies showed that risk of death in patients with CKD (Figure 2D) was higher than for HT and DM (OR = 3.91, 95% CI: 2.50–6.10 and *p*-value  < 0.001). The *I*^2^ test results for each secondary outcome revealed that there was considerable heterogeneity with *I*^2^  =  84.89% for HT, 88.91% for DM and 92.39% for CKD.

For HT data, Egger’s meta-regression test did not reveal significant effects of small studies (*β*_1_ = 0.14, SE *β*_1_ = 0.69, *z* = 0.21, *p* = 0.8353), indicating no evidence of publication bias in the analyzed group of 14 studies. Despite high heterogeneity (*I*^2^ = 84.89%), no systematic asymmetry related to study precision was observed. 

For the data defining DM, the Egger’s test based on meta-regression did not reveal significant effects of small studies (*β*_1_ = 1.171, SE *β*_1_ = 0.657, *z* = 1.78, *p* = 0.0746), indicating no evidence of publication bias in the analyzed group of 15 studies. Despite the high heterogeneity (*I*^2^ = 88.91%), no systematic asymmetry related to study precision was observed, but it is a warning signal. 

For the data defining CKD, the Egger’s test based on meta-regression did not reveal significant effects of small studies (*β*_1_ = 0.32, SE = 0.848, *z* = 0.38, *p* = 0.7051), indicating no evidence for the presence of publication bias in the analyzed group of 12 studies. Despite high heterogeneity (*I*^2^ = 92.39%), no systematic asymmetry related to the precision of studies was observed.

The funnel plot (Figure 3) and Egger’s test revealed no evidence of small-study effects or publication bias across all subgroups, with the exception of the DM subgroup, in which a weak trend towards small-study effects was noted; however, this did not reach statistical significance (*p* = 0.0746).

**Figure 4:**
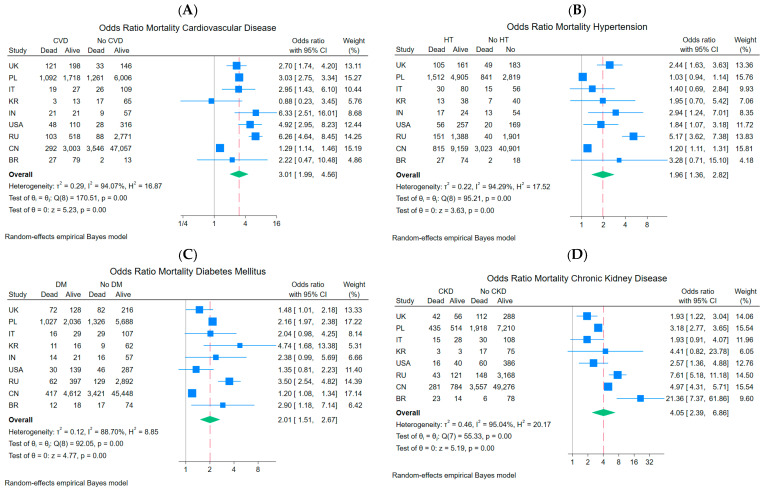
Forest plot of mortality rate for patients with vs. without: (**A**), cardiovascular disease; (**B**), hypertension; (**C**), diabetes mellitus; (**D**), chronic kidney disease depending on the country of the study showing the pooled odds ratio (OR) with 95% confidence intervals (95% CIs).

In the geographic analysis of CVD, the highest ORs were observed in studies from India (OR = 6.33, 95% CI: 2.51–16.01) and Russia (OR = 6.26, 95% CI: 4.64–8.45), which may reflect limited access to specialized medical care, delayed diagnosis, or inadequate control of cardiovascular risk factors (Figure 4A). In contrast, the study from Brazil showed a lower risk (OR = 2.22, 95% CI: 0.47–10.48) with a wide confidence interval, which may be due to greater clinical heterogeneity among patients. Despite the variation between studies, all OR estimates were greater than 1, and most of them reached statistical significance, strengthening the conclusion that CVD is an independent risk factor for mortality in COVID-19. Furthermore, the weighting of individual studies varied significantly, with large samples from Russia, China, and Poland having the greatest impact on the final result. This may lead to data from these populations dominating the pooled estimate relative to smaller studies, which should be taken into account when interpreting the pooled results. In each case, the vast majority of studies from different countries showed a positive association between a given comorbidity and the risk of death in COVID-19 (Figure 4A–D). Only a few studies, e.g., PL for HT: OR = 1.03, 95% CI: 0.94–1.14 (Figure 4B), did not reach statistical significance, which may be related to large sample sizes, different patient characteristics, or more thorough treatment of comorbidities.

**Figure 5:**
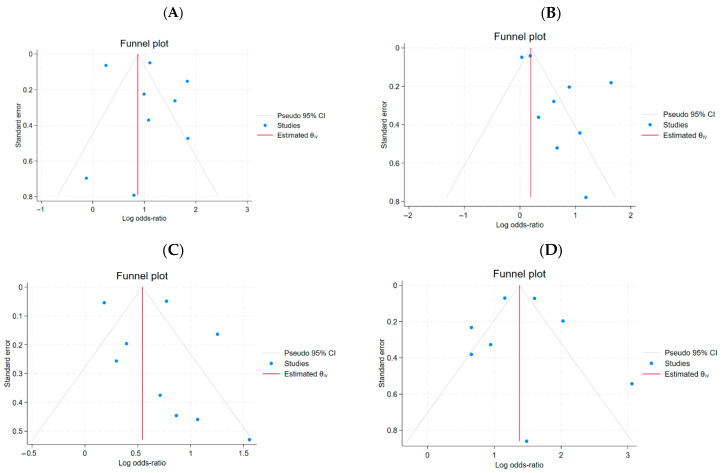
Funnel plot of publication bias analysis for studies depending on the country included: (**A**), cardiovascular disease; (**B**), hypertension; (**C**), diabetes mellitus; (**D**), chronic kidney disease.

In the country-level analysis, the funnel plot results and Egger’s meta-regression test (random-effects method, Empirical Bayes) did not reveal significant effects of small studies for CVD (*β*_1_ = −0.29; SE *β*_1_ = 1.052, *z* = −0.28, *p* = 0.7817), HT (*β*_1_ = 1.13; SE *β*_1_ = 0.971, *z* = 1.17, *p* = 0.2427), DM (*β*_1_ = 1.19; SE *β*_1_ = 0.896, *z* = 1.33, *p* = 0.1827) or CKD data (*β*_1_ = 0.75; SE *β*_1_ = 1.355, *z* = 0.55, *p* = 0.5816), indicating no evidence of publication bias in the analyzed group of 9 countries (Figure 5). 

In the geographic subgroup analysis, despite high heterogeneity (I^2^ = 94.07%, 94.29%, 88.70%, and 95.04% for CVD, HT, DM, and CKD, respectively), no systematic asymmetry related to study precision was observed.

## 4. Discussion

This systematic review with meta-analysis assessed the association between mortality among patients infected with COVID-19 and four key comorbidities: CVD, HT, DM, and CKD. The meta-analysis was conducted using data from 68,963 patients from 15 cohort studies across nine countries. 

The results of our analysis clearly indicate that pre-existing comorbidities significantly increase the risk of death during COVID-19 infection. The highest cumulative mortality was observed in patients with CKD (35.5%), followed by CVD (23.3%), DM (18.3%) and HT (16.3%). 

The overall mortality rate observed in our pooled cohort (9.7%; 6736/68,963) is consistent with estimates reported in other large meta-analyses covering comparable pandemic phases, although considerable geographic variation is apparent across the included studies (Table 2). The Polish cohort—the largest geographically homogeneous subgroup in our dataset, aggregating 10,077 patients across three studies—demonstrated a pooled mortality of 23.4% (2353/10,077), substantially exceeding the rates observed in the Russian (5.5%; 191/3480), Chinese (7.1%; 3838/53,898), and American (15.1%; 76/502) cohorts. This variation likely reflects differences in healthcare system capacity, patient age structure, comorbidity burden, and the timing of each cohort relative to pandemic waves and warrants cautious interpretation of any pooled estimate.

### 4.1. Cardiovascular Disease as a Risk Factor for Death

Patients with CVD were three times more likely to die from COVID-19 compared with patients without CVD (OR = 3.00; 95% CI: 2.06–4.38). Despite significant heterogeneity between studies (*I*^2^ = 93.66%), Egger’s test did not reveal a significant effect of small studies, which confirms the reliability of the observed association. The rationale for this observation is multifaceted. SARS-CoV-2 enters host cells via angiotensin-converting enzyme 2 (ACE2), which is highly expressed in cardiomyocytes, vascular endothelium and renal tubular epithelial cells [25]. Binding of the virus to ACE2 leads to internalization and subsequent downregulation of this receptor. This disrupts the ACE2/angiotensin-(1–7)/mitochondrial assembly receptor (ACE2/Ang-(1–7)/MasR) axis—the counter-regulatory arm of the renin–angiotensin–aldosterone system (RAAS), which inhibits hypertensive and tissue-damaging processes. The result of this disruption is the uncontrolled accumulation of angiotensin II, which leads to oxidative stress, endothelial dysfunction, vasoconstriction and severe inflammation, including of the myocardium [26]. In patients with pre-existing CVD, the RAAS system is already chronically dysregulated; therefore, viral ACE2 depletion superimposed on this condition triggers a disproportionately severe pathological response [27]. This mechanism directly explains why cardiovascular diseases are a significant factor increasing the risk of mortality. At the myocardial level, SARS-CoV-2 exerts a direct cytopathic effect on cardiomyocytes and cardiac pericytes, inducing mitochondrial dysfunction, impaired oxidative phosphorylation, and activation of the intrinsic apoptotic cascade through the release of cytochrome c and activation of caspase-3 [28]. Simultaneously, a systemic cytokine storm characterized by a massive increase in serum interleukins (IL) such as IL-6 and IL-1β, tumor necrosis factor-alpha (TNF-α), and interferon-gamma (IFN-γ) concentrations exacerbates myocardial inflammation via the signal transducer and activator of transcription 3 (STAT3) and mitogen-activated protein kinase/extracellular signal-regulated kinase (MAPK/ERK) signaling pathways, leading to acute myocarditis, stress cardiomyopathy, and arrhythmogenicity [29]. In patients with structural heart disease, these mechanisms immediately translate into hemodynamic destabilization. The risk of thrombotic complications and organ failure (heart, kidneys) may be increased through endothelial and microcirculation damage [30,31]. This explains the disproportionately high increase in mortality in this cohort.

Our OR of 3.00 (95% CI: 2.06–4.38) for CVD-associated mortality is broadly consistent with, though modestly lower than, estimates from comparable meta-analyses. Figliozzi et al. reported an OR of 3.15 (95% CI: 2.26–4.41) for in-hospital mortality in COVID-19 patients with pre-existing CVD across 11 studies (N = 12,717) [32]. Similarly, Ssentongo et al. identified a pooled risk ratio of 2.25 (95% CI: 1.53–3.29) for mortality associated with cardiac disease in a meta-analysis of 14 studies comprising 2463 patients [33]. The modest shift in our estimate relative to these earlier analyses likely reflects the inclusion of larger, population-level cohorts from the later pandemic period—particularly He et al. (2025, N = 53,030) [22] and Munblit et al. (2021, N = 3480) [20]—in which improved clinical management of COVID-19 in patients with cardiovascular disease may have attenuated mortality risk compared to the earliest pandemic wave. Notably, CVD prevalence in our cohort (10.7%; 7413/68,963) was lower than the 15.1% reported by Ssentongo et al. [33], which may additionally reflect differences in case ascertainment across the wider geographic and temporal scope of our analysis.

### 4.2. Arterial Hypertension: The Most Common but Not the Most Dangerous Comorbidity

Although HT was the most common comorbidity (27.3% of the study population), it was associated with a lower risk of death (OR = 1.94; 95% CI: 1.50–2.52) compared with CVD or CKD. There was no evidence of publication bias (Egger’s test *p* = 0.8353). The lower odds ratio may be due to the effectiveness of antihypertensive treatment in different populations and the different age structure of patients with hypertension. However, the pathophysiology of this association is important: chronic hypertension leads to chronic endothelial dysfunction and RAAS overactivity [34]. Vascular shear stress, characteristic of hypertension, damages the endothelial glycocalyx, facilitating viral adhesion, activation of the coagulation cascade, and the development of thrombotic microangiopathy [30,35]. However, unlike CVD and CKD, hypertension per se (in itself) is not associated with an equally profound impairment of innate immunity or with comparable direct organ damage, which may explain its moderate impact on mortality.

The OR of 1.94 (95% CI: 1.50–2.52) observed in our analysis for hypertension-associated mortality is lower than in several earlier meta-analyses. Pranata et al. reported an OR of 2.21 (95% CI: 1.74–2.81) for mortality in hypertensive COVID-19 patients across 30 studies (N = 6560) [36], while a meta-analysis by Lippi et al. identified a pooled OR of 2.42 (95% CI: 1.51–3.90) across 13 studies (N = 2842) [37]. The attenuation observed in our estimate may partly reflect the high prevalence of treated hypertension in the large Eastern European cohorts (PL: 6417/10,077; 63.7%), where antihypertensive therapy—particularly RAAS inhibitors—may have conferred a degree of endothelial protection during acute infection. This interpretation is consistent with evidence suggesting that ACE inhibitor and angiotensin receptor blocker use is not associated with adverse outcomes in COVID-19 and may be beneficial in certain subgroups [38].

### 4.3. Diabetes: Metabolic Immunosuppression and Oxidative Stress

Patients with DM also had a significantly increased risk of death (OR = 2.17; 95% CI: 1.64–2.86). Although Egger’s test (*p* = 0.0746) did not reach statistical significance, it indicated a potential trend toward a small-study effect, which warrants caution in this subgroup. Diabetes worsens the prognosis in COVID-19 through multifaceted mechanisms. Hyperglycemia increases oxidative stress, activation of the nuclear factor kappa-light-chain-enhancer of activated B cell (NF-κB) pathway, and production of proinflammatory cytokines [39,40]. At the same time, it impairs the immune response by impairing neutrophil and T-cell function [41]. Diabetes also promotes glycation of receptor proteins, which may alter the interaction of the virus with ACE2 and exacerbate endothelial dysfunction and coagulopathy [42]. Diabetic micro- and macroangiopathy, a complication of chronic hyperglycemia, creates a vascular substrate particularly susceptible to viral endothelial damage, leading to a pathological vicious cycle of inflammation, thrombosis, and organ damage [31,40].

Our pooled OR of 2.17 (95% CI: 1.64–2.86) for diabetes-associated mortality is consistent with estimates from high-quality meta-analyses published in peer-reviewed journals with impact factors. Huang et al. reported a pooled RR of 2.12 (95% CI: 1.44–3.11) for mortality in diabetic COVID-19 patients across 30 studies (N = 6452) [43]. A larger analysis by Kumar et al. encompassing 33 studies and 16,003 patients yielded a pooled OR of 1.90 (95% CI: 1.37–2.64) [44]. The borderline small-study effect observed in our Egger’s test (*p* = 0.0746) likely reflects the inclusion of smaller single-center cohorts (N = 108 [14]; N = 26 [16]; N = 98 [17]) with DM mortality estimates disproportionately influenced by individual outcomes. Notably, DM prevalence in our cohort (13.1%; 9057/68,963) and DM-associated crude mortality (18.3%) closely mirrors the figures reported by the Chinese national surveillance cohort included in our analysis (DM N = 4922; DM dead N = 387 [22]), suggesting that the aggregate estimate is predominantly driven by this large dataset.

### 4.4. Chronic Kidney Disease: The Strongest Predictor of Mortality

Among all comorbidities analyzed, CKD was associated with the highest risk of death (OR = 3.91; 95% CI: 2.50–6.10). This result highlights the extreme susceptibility of patients with impaired renal function to SARS-CoV-2 infection. Despite significant heterogeneity (*I*^2^ = 92.39%), Egger’s test did not confirm publication bias. The particularly high risk observed in CKD reflects a distinct, overlapping cascade of mechanisms. ACE2 expression in the glomeruli and tubules is the highest among all organ systems, making the kidneys a primary site of direct tropism for SARS-CoV-2 [45]. Virus-induced acute kidney injury (AKI) in patients with CKD is superimposed on an already severely reduced nephron mass, triggering a rapid decline in residual functional reserve [46]. Furthermore, CKD-associated chronic inflammation, complement dysregulation, and impaired neutrophil and monocyte function create an immunologically permissive environment for uncontrolled viral replication and cytokine storm [47]. The resulting accumulation of uremic toxins—including indoxyl sulfate and p-cresol sulfate—directly inhibits mitochondrial function in cardiomyocytes and hepatocytes, leading to rapid progression toward multiple organ failure [48]. The cardiorenal axis, in which cardiac dysfunction reduces renal perfusion and renal failure exacerbates cardiac volume and pressure overload, is a central mechanism underlying the synergistic mortality risk observed in the presence of CKD [49].

The OR of 3.91 (95% CI: 2.50–6.10) observed in our analysis for CKD represents the highest estimate among all four comorbidities examined and is notably higher than estimates reported in earlier meta-analyses, likely genuinely reflecting the cardiorenal synergy mechanisms described above. Cai et al. reported an OR of 5.81 (95% CI: 3.78–8.94) for mortality in CKD patients with COVID-19 across 12 studies (N = 3,867,367) [50]; this higher estimate may reflect the earlier pandemic period (predominance of ancestral strain and limited critical care capacity) and smaller constituent cohorts. Cheng et al. documented that AKI occurred in 36.6% of hospitalized COVID-19 patients and was independently associated with in-hospital mortality (HR = 5.33; 95% CI: 3.40–8.36), reinforcing the central role of the renal axis in COVID-19 fatality [51]. In our pooled dataset, CKD-associated crude mortality reached 35.5% (858/2418), a figure markedly exceeding that of any other comorbidity subgroup and consistent with the disproportionate nephron vulnerability described above. The high heterogeneity (*I*^2^ = 92.39%) observed in this subgroup likely reflects actual differences in baseline CKD severity (stage 3 vs. 4–5) and dialysis dependence across the included cohorts, none of which reported data on CKD severity.

### 4.5. Pathological Convergence: A Common Final Pathway When Comorbidities Overlap

When the analyzed comorbidities coexist in a single patient, their effects appear to converge at the level of the vascular endothelium. Hyperglycemia, shear stress associated with hypertension, uremic toxins, and direct SARS-CoV-2 infection each independently damage the endothelial glycocalyx, promoting vascular microthrombosis, disseminated intravascular coagulation (DIC), and systemic endotheliitis [52]. Mechanistically, the synergistic increase in mortality risk with the coexistence of CVD, CKD, DM, and HT is explained by the convergence of at least four independent pathological axes: (1) RAAS dysregulation and excess angiotensin II; (2) degradation of the endothelial glycocalyx, facilitating DIC and microthrombosis via upregulation of tissue factor and release of von Willebrand factor; (3) impaired innate immunity secondary to uremia and hyperglycemia; and (4) mitochondrial bioenergetic failure simultaneously in multiple organ systems [53]. The intersection of these axes at the level of the vascular endothelium—itself a primary target of SARS-CoV-2—transforms localized pulmonary infection into systemic endothelitis, constituting the histopathological picture of multiorgan failure. In this context, the ORs observed for individual comorbidities almost certainly underestimate the true synergistic risk of mortality in patients presenting with full cardiometabolic–renal burden.

The convergence hypothesis advanced above is supported by population-level data from our analysis. In the Polish cohort—for which comorbidity co-occurrence data are most completely reported across the three constituent studies [11,12,13]—the proportion of patients carrying at least two of the four analyzed comorbidities simultaneously exceeded 40%, consistent with the well-established epidemiological clustering of cardiometabolic conditions. This synergistic amplification is mechanistically coherent with the convergent endothelial damage pathways described in this section and further supports the clinical relevance of integrated cardiometabolic–renal risk stratification.

### 4.6. Impact of SARS-CoV-2 Strain Variation on Pathogenicity and Clinical Outcomes

The studies included in this analysis mainly reflect the early phase of the pandemic dominated by the ancestral Wuhan strain (D614G) and the Alpha variant (B.1.1.7), with partial overlap with the early Delta wave (B.1.617.2) in the largest cohort (through August 2021) [22]. Although during this period the pathophysiological relationship between cardiovascular comorbidities and COVID-19 mortality was least affected by vaccination and antiviral therapy, our initial intention was to cover the entire pandemic period. Systematic database searches across August 2020 to June 2025 revealed no eligible studies with observational periods extending beyond 2021, reflecting the nature of available evidence rather than a selection bias.

The Wuhan, Alpha, and Delta variants share a pathophysiological profile characterized by pronounced endothelial dysfunction, systemic hypercoagulability, and hyperinflammation [54,55]—mechanisms directly underlying the adverse outcomes observed in patients with pre-existing CVD, HT, DM, and CKD. In patients with pre-existing endothelial compromise, this viral-induced endotheliopathy is further amplified, likely explaining the disproportionate mortality burden documented across our pooled cohort.

The epidemiological landscape shifted substantially from late 2021 onward with the emergence of Omicron (B.1.1.529) and its sublineages, which demonstrate a tropism shift towards the upper respiratory tract, markedly lower replication in lung parenchymal tissue [56,57], and intrinsically reduced capacity to induce endothelial injury and coagulopathy [58]. Meta-analytic evidence confirms approximately 61% lower risk of death with Omicron compared to Delta [59]. Emerging data further suggest that the prognostic burden of cardiovascular comorbidities may be attenuated in the Omicron era, partly owing to vaccination-mediated reduction in severe cardiovascular events [60,61].

The conclusions of this analysis can therefore be most accurately applied to the pre-vaccination and pre-Omicron-dominated pandemic phases. Future studies stratifying outcomes by confirmed variant type are needed to determine whether the association between CVD, HT, DM, and CKD and COVID-19 mortality persisted, weakened, or changed across viral lineages.

### 4.7. Comparison with Previous Meta-Analyses

A similar previous systematic review [62] identified 307,596 patients from 16 reports published up to 25 July 2020, in which 46,321 patients (15.1%) had cardiovascular disease (in our study 10.7%, i.e., 7413). The overall mortality rate in that study was similar to ours: 8.2% (20,534 patients) versus 9.7% (6736) in our analysis. Comparable results were also obtained for mortality in patients with CVD—a total of 24.2% (11,213) of patients with CVD died [62], compared to 23.3% (1726) in our study. Cardiovascular disease in that study was associated with a four-fold increased risk of death (OR = 4.33; 95% CI: 3.16–5.94), whereas in our analysis we observed a three-fold increase (OR = 3.0; 95% CI: 2.06–4.38). This difference is likely due to the inclusion of cases from the later period of the pandemic in our meta-analysis, when care for at-risk patients had improved somewhat. Similarly, diabetes in the comparator study [62] was associated with an approximately 2.5-fold increased risk of death (OR = 2.41; 95% CI: 1.79–3.26), which was slightly higher than in our analysis (OR = 2.17; 95% CI: 1.64–2.86). Hypertension also showed a higher odds ratio in the older study (OR = 2.60; 95% CI: 2.10–3.21) [62] compared to our analysis (OR = 1.94; 95% CI: 1.50–2.52), although this difference was not statistically significant. Despite the existence of earlier studies, our meta-analysis incorporates new data from the later pandemic period and includes a regional subgroup of Eastern European populations, providing new information on geographic variation and risk estimates.

The consistent downward trend in OR estimates across all four comorbidities when comparing our analysis to the earlier meta-analysis [46]—published exclusively on data from the first pandemic wave—deserves explicit commentary. This systematic attenuation is unlikely to reflect methodological differences alone. It is most parsimoniously explained by three concurrent factors: (1) improved in-hospital management of COVID-19 in patients with cardiometabolic comorbidities across the 2020–2021 period, including earlier anticoagulation, corticosteroid therapy, and targeted organ support; (2) the inclusion of large Eastern European cohorts with systematically documented comorbidities and standardized mortality ascertainment, which may reduce the ascertainment bias in early single-center Asian case series; and (3) the natural enrichment of the later-pandemic study population with patients who had milder disease courses as severe early cases were disproportionately represented in the initial literature. This temporal attenuation of comorbidity-associated mortality risk is consistent with findings from the OpenSAFELY cohort (N > 17 million) [63], which demonstrated a progressive reduction in the excess mortality associated with pre-existing conditions as clinical protocols matured during the first pandemic year.

### 4.8. Heterogeneity and Publication Bias

Although all results showed high heterogeneity (*I*^2^ > 84%), most likely reflecting differences in study populations, comorbidity definitions, and healthcare settings, no significant publication bias was observed in any subgroup (Egger’s tests and funnel plot analysis). Importantly, the geographic origin of the studies (nine countries) did not influence the pattern of bias, and analysis stratified by country confirmed the robustness of the pooled estimates. The funnel plot results (Figure 3 and Figure 5) and Egger’s tests did not reveal a small study effect, suggesting a lack of evidence of publication bias. Only in the diabetes subgroup was a weak trend indicating a small study effect observed; however, it did not reach statistical significance (*p* = 0.0746). 

The high heterogeneity observed across all four comorbidity subgroups (*I*^2^ range 84–94%) is a recognized feature of COVID-19 meta-analyses and has been documented consistently in the literature. Borges do Nascimento et al. observed *I*^2^ values exceeding 80% in virtually all subgroup analyses of a 212-study meta-analysis on COVID-19 outcomes (N = 281,461), attributing this to the marked variation in healthcare system responses, case ascertainment methods, and comorbidity coding practices across countries and pandemic phases [64]. In our analysis, the geographic stratification presented in Table 2 provides partial insight into the sources of this heterogeneity: the Chinese cohort (N = 53,898; mortality 7.1%) and the Polish cohort (N = 10,077; mortality 23.4%) represent the two largest geographic subgroups and display a three-fold difference in crude mortality, reflecting genuine population-level differences in patient age (median 58 vs. 63 years), comorbidity clustering, and healthcare capacity rather than methodological artefact. This observation reinforces the importance of region-specific risk stratification and cautions against uncritical global extrapolation of pooled OR estimates.

### 4.9. Limitations

This systematic review has some limitations. First, 15 studies were included, in which the majority of patients were hospitalized, ensuring some homogeneity of the cohort. However, the severity of COVID-19 and the immune status of the patients (vaccination history) were unknown and could not be included in the analysis. Publication and selection bias cannot be completely excluded as data may have been published primarily on patients with atypical characteristics and poor prognosis. The residual variability (heterogeneity) assessment tool has limitations, particularly when small sample sizes and studies differing in time, location, and broad inclusion criteria were included in the analysis. Subgroup analyses by geographic location and the exclusion of small study groups partially explain the sources of high heterogeneity, but the primary goal of this study was to estimate the mean mortality rate for the entire population. Furthermore, meta-analytically combining heterogeneous study populations does not allow for precise estimation of the synergistic risk of mortality resulting from the overlap of multiple comorbidities—this is a limitation of this research method. 

A limitation of this study is the absence of a prospectively registered protocol (e.g., in PROSPERO). However, the review was conducted in accordance with PRISMA guidelines, which partially mitigates concerns regarding methodological transparency.

A significant limitation of this analysis is the heterogeneity of sample sizes across the included studies. Several cohorts contain fewer than 500 patients, which limits the individual representativeness of these datasets, and conclusions drawn from them should be very cautious. The inclusion of smaller studies was motivated by the need to ensure geographic diversity and capture early clinical patterns of the pandemic in underrepresented regions. Because pooled analysis is strongly biased towards large-scale studies, the three largest cohorts (N = 53,030 [22]; N = 3480 [20]; N = 4277 [11]) together represent approximately 88% of the entire study population; the small sample sizes did not affect the pooled estimates and conclusions drawn from them.

Several additional limitations merit explicit acknowledgement. First, none of the included studies reported CKD staging (GFR categories G1–G5) or dialysis dependence, precluding a severity-stratified analysis of renal function and COVID-19 mortality—a clinically important distinction given that dialysis-dependent patients carry substantially higher baseline mortality risk [50]. Second, comorbidity definitions varied across studies: some relied on ICD-coded discharge diagnoses, others on physician-reported history at admission, creating potential misclassification that would tend to dilute true effect sizes. Third, the absence of data on concurrent comorbidity combinations across all 15 studies prevents a formal interaction analysis, and the synergistic mortality risk for patients carrying all four comorbidities simultaneously can only be inferred indirectly from the convergence mechanisms described in Section 4.5. Finally, data on vaccination or specific pharmacological treatments received during hospitalization—anticoagulation, corticosteroids, remdesivir, tocilizumab—are not reported in any of the included studies, limiting the ability to assess whether treatment heterogeneity contributed to the variation in observed mortality rates across cohorts and countries.

## 5. Conclusions

This meta-analysis highlights a strong and independent association between major comorbidities and increased mortality in COVID-19 patients, with CKD and CVD being the most important risk factors. CKD increases the risk of COVID-19-related death by nearly fourfold and CVD by threefold. Moreover, comorbidities such as HT and DM also significantly increase the risk of death in people infected with SARS-CoV-2 by two times. 

The analysis confirms that chronic diseases are key risk factors for an unfavorable course of COVID-19, which is consistent with previous reports from, among others, WHO, CDC, and the results of large multi-center analyses. Public health strategies should prioritize vaccination and monitoring of individuals with these conditions. There is still a need to develop healthcare strategies that reduce the risk of death for patients with cardiovascular disease in situations such as the COVID-19 pandemic.

## Figures and Tables

**Table 1:** Characteristics of included published studies.

Study Reference	Country	N Total	N Dead	Median Age (IQR), y	Male	CVD	CVD Dead	HT	HT Dead	DM	DM Dead	CKD	CKD Dead	Observational Period
Khawaja et al., 2021 [10]	UK	498	154	67.4 ± 16.1 ^a^	310	319	121	266	105	200	72	98	42	7 March 2020−7 April 2020
Gasior et al., 2021 [11]	PL	4277	1794	72.0 ^a^	2426	1947	872	3153	1355	1629	762	669	360	March 2020−December 2020
Trytko et al., 2025 [12]	PL	4071	336	59.7 ^a^	2183	573	153	2294	Nd	989	181	280	75	13 February 2020−10 May 2021
Terlecki et al., 2021 [13]	PL	1729	223	63.0 (50–75)	886	290	67	970	157	445	84	nd	nd	6 March 2020−15 October 2020
Bensai et al., 2021 [14]	IT	108	24	71.0	78	22	9	65	18	33	8	24	7	1 October 2020−31 December 2020
Bottio et al., 2021 [15]	IT	47	14	61.8 ± 14.51 ^a^	37	9	6	30	8	8	5	6	2	February 2020
Iavaconi et al., 2020 [16]	IT	26	7	63.0 (58−72)	20	15	4	15	4	4	3	13	6	February 2020−March 2020
Lee et al., 2020 [17]	KR	98	20	72.0 (68–79)	44	16	3	51	13	27	11	6	3	18 February 2020−4 March 2020
Kunal et al., 2020 [18]	IN	108	30	51.2 ± 17.7 ^a^	70	42	21	41	17	35	14	nd	nd	nd
Hobbs et al., 2021 [19]	USA	502	76	62.0 (49–71)	277	158	48	313	56	169	30	56	16	1 March 2020−8 May 2020
Munblit et al., 2021 [20]	RU	3480	191	56.0 (45–66)	1758	621	103	1539	151	459	62	164	43	8 April 2020−28 May 2020
Liu et al., 2021 [21]	CN	665	70	58.0 (47–68)	318	57	22	158	27	78	19	20	8	1 January 2020−4 March 2020
He et al., 2025 [22]	CN	53,030	3710	59.0 (47–70)	26,546	3213	260	9736	759	4922	387	1045	273	29 December 2019−31.08.2021
Yang et al., 2021 [23]	CN	203	58	62.0 (49–69)	115	25	10	80	29	29	11	nd	nd	10 January 2020−29 February 2020
Paulino et al., 2021 [24]	BR	121	29	64.0 (33–72)	80	106	27	101	27	30	12	37	23	March 2020−October 2020
Overall		68,963	6736	>51.0	35,148	7413	1726	18,812	3071	9057	1661	2418	858	29 December 2019−31 August 2021

IQR—interquartile range, CVD—cardiovascular disease, HT—hypertension, DM—diabetes mellitus, CKD—chronic kidney disease, nd—no data, UK—United Kingdom, PL—Poland, IT—Italy, KR—South Korea, IN—India, USA—United States of America, RU—Russia, CN—China, BR—Brazil. ^a^—Arithmetic mean ± standard deviation.

**Table 2:** Geolocation characteristics of published studies.

Country	N Total	N Dead	Median Age (IQR), y	Male	CVD	CVD Dead	HT	HT Dead	DM	DM Dead	CKD	CKD Dead	Observational Period
UK [10]	498	154	67.4 ± 16.1 ^a^	310	319	121	266	105	200	72	98	42	7 March 2020–7 April 2020
PL [11,12,13]	10,077	2353	63.0 (50–75)	5495	2810	1092	6417	1512	3063	1027	949	435	13 February 2020–10 May 2021
IT [14,15,16]	181	45	63.0 (58−72)	135	46	19	110	30	45	16	43	15	February 2020–31 December 2020
KR [17]	98	20	72.0 (68–79)	44	16	3	51	13	27	11	6	3	18 February 2020–4 March 2020
IN [18]	108	30	51.2 ± 17.7 ^a^	70	42	21	41	17	35	14	nd	nd	nd
USA [19]	502	76	62.0 (49–71)	277	158	48	313	56	169	30	56	16	1 March 2020–8 May 2020
RU [20]	3480	191	56.0 (45–66)	1758	621	103	1539	151	459	62	164	43	8 April 2020–28 May 2020
CN [21,22,23]	53,898	3838	58.0 (47–70)	26,979	3295	292	9974	815	5029	417	1065	281	29 December 2019–31 August 2021
BR [24]	121	29	64.0 (33–72)	80	106	27	101	27	30	12	37	23	March 2002–October 2020
Overall	68,963	6736	>51.0	35,148	7413	1726	18,812	3071	9057	1661	2418	858	29 December 2019–31 August 2021

IQR—interquartile range, CVD—cardiovascular disease, HT—hypertension, DM—diabetes mellitus, CKD—chronic kidney disease, nd—no data, UK—United Kingdom, PL—Poland, IT—Italy, KR—South Korea, IN—India, USA—United States of America, RU—Russia, CN—China, BR—Brazil. ^a^—Arithmetic mean ± standard deviation.

## Data Availability

The relevant data are available from M.J. upon reasonable request.

## Supplementary Materials

The following supporting information can be downloaded at: https://www.mdpi.com/article/10.3390/ijms27104375/s1. Ref. [65] is cited in the Supplementary Materials.

## References

[1] WHO (2024). WHO Coronavirus Disease (COVID-19) Dashboard.

[2] Msemburi W., Karlinsky A., Knutson V., Aleshin-Guendel S., Chatterji S., Wakefield J. (2023). The WHO Estimates of Excess Mortality Associated with the COVID-19 Pandemic. Nature.

[3] Walory J., Ksiazek I., Karynski M., Baraniak A. (2023). Twenty-Month Monitoring of Humoral Immune Response to BNT162b2 Vaccine: Antibody Kinetics, Breakthrough Infections, and Adverse Effects. Vaccines.

[4] Walory J., Ksiazek I., Wegrzynska K., Baraniak A. (2024). Comparison of Post-Vaccination Response (Humoral and Cellular) to BNT162b2 in Clinical Cases, Kidney and Pancreas Transplant Recipient with Immunocompetent Subjects over Almost Two Years of Parallel Monitoring. Vaccines.

[5] Komiazyk M., Walory J., Gawor J., Ksiazek I., Gromadka R., Baraniak A. (2021). Case Report of COVID-19 after Full Vaccination: Viral Loads and Anti-SARS-CoV-2 Antibodies. Diagnostics.

[6] Zhang T., Li Z., Mei Q., Walline J.H., Zhang Z., Liu Y., Zhu H., Du B. (2025). Cardiovascular Outcomes in Long COVID-19: A Systematic Review and Meta-Analysis. Front. Cardiovasc. Med..

[7] Momtazmanesh S., Shobeiri P., Hanaei S., Mahmoud-Elsayed H., Dalvi B., Malakan Rad E. (2020). Cardiovascular Disease in COVID-19: A Systematic Review and Meta-Analysis of 10,898 Patients and Proposal of a Triage Risk Stratification Tool. Egypt Heart J..

[8] Haddaway N.R., Page M.J., Pritchard C.C., McGuinness L.A. (2022). *PRISMA2020*: An R Package and Shiny App for Producing PRISMA 2020-compliant Flow Diagrams, with Interactivity for Optimised Digital Transparency and Open Synthesis. Campbell Syst. Rev..

[9] StataCorp LLC (2023). Stata Statistical Software.

[10] Khawaja S.A., Mohan P., Jabbour R., Bampouri T., Bowsher G., Hassan A.M.M., Huq F., Baghdasaryan L., Wang B., Sethi A. (2021). COVID-19 and Its Impact on the Cardiovascular System. Open Heart.

[11] Gąsior M., Jaroszewicz J., Wita K., Cieśla D., Hudzik B. (2021). High Post-Discharge Mortality in Hospitalized COVID-19 Patients with Cardiovascular Comorbidities-. Pol. Arch. Intern. Med..

[12] Kocowska-Trytko M., Terlecki M., Olszanecka A., Pavlinec C., Rajzer M., Adamczyk M., Andrychiewicz A., Antczak J., Banaszkiewicz M., CraCov HHS Investigators (2025). Sex and Other Predictors of Mortality in Long-Term Follow-up of Patients with Cardiovascular Disease and COVID-19: A Single-Center Retrospective Study. Sci. Rep..

[13] Terlecki M., Wojciechowska W., Klocek M., Olszanecka A., Stolarz-Skrzypek K., Grodzicki T., Małecki M., Katra B., Garlicki A., Bociąga-Jasik M. (2021). Association between Cardiovascular Disease, Cardiovascular Drug Therapy, and in-Hospital Outcomes in Patients with COVID-19: Data from a Large Single-Center Registry in Poland. Kardiol. Pol..

[14] Bensai S., Oldani S., Bertolovic L., Colinelli C., Simoncelli S., Ghirotti C., Agnoletti V., Maitan S., Mezzatesta L., Valpiani G. (2022). Experience of the Second Wave of COVID-19 in Italy: A Cohort Study in a Respiratory Semi-Intensive Care Unit. Rass. Patol. Dell’Apparato Respir..

[15] Bottio T., Bagozzi L., Fiocco A., Nadali M., Caraffa R., Bifulco O., Ponzoni M., Lombardi C.M., Metra M., Russo C.F. (2021). COVID-19 in Heart Transplant Recipients. JACC Heart Fail..

[16] Iacovoni A., Boffini M., Pidello S., Simonato E., Barbero C., Sebastiani R., Vittori C., Fontana A., Terzi A., De Ferrari G.M. (2020). A Case Series of Novel Coronavirus Infection in Heart Transplantation from 2 Centers in the Pandemic Area in the North of Italy. J. Heart Lung Transplant..

[17] Lee J.Y., Kim H.A., Huh K., Hyun M., Rhee J.-Y., Jang S., Kim J.-Y., Peck K.R., Chang H.-H. (2020). Risk Factors for Mortality and Respiratory Support in Elderly Patients Hospitalized with COVID-19 in Korea. J. Korean Med. Sci..

[18] Kunal S., Sharma S.M., Sharma S.K., Gautam D., Bhatia H., Mahla H., Sharma S., Bhandari S. (2020). Cardiovascular Complications and Its Impact on Outcomes in COVID-19. Indian Heart J..

[19] Hobbs A.L.V., Turner N., Omer I., Walker M.K., Beaulieu R.M., Sheikh M., Spires S.S., Fiske C.T., Dare R., Goorha S. (2021). Risk Factors for Mortality and Progression to Severe COVID-19 Disease in the Southeast Region in the United States: A Report from the SEUS Study Group. Infect. Control Hosp. Epidemiol..

[20] Munblit D., Nekliudov N.A., Bugaeva P., Blyuss O., Kislova M., Listovskaya E., Gamirova A., Shikhaleva A., Belyaev V., Timashev P. (2021). Stop COVID Cohort: An Observational Study of 3480 Patients Admitted to the Sechenov University Hospital Network in Moscow City for Suspected Coronavirus Disease 2019 (COVID-19) Infection. Clin. Infect. Dis..

[21] Liu M., Han S., Liao Q., Chang L., Tan Y., Jia P., Yang L., Cai H., Feng S., Chen C. (2021). Outcomes and Prognostic Factors in 70 Non-survivors and 595 Survivors with COVID-19 in Wuhan, China. Transbound. Emerg. Dis..

[22] He W., Li G., Xu K., Yu B., Sun Y., Zhong K., Zhou D., Yan Y., Wu J., Wang D.W. (2025). Clinical Characteristics and Risk Factors for In-Hospital Mortality of COVID-19 Patients in Hubei Province: A Multicenter Retrospective Study. Int. J. Cardiol. Heart Vasc..

[23] Yang C., Liu F., Liu W., Cao G., Liu J., Huang S., Zhu M., Tu C., Wang J., Xiong B. (2021). Myocardial Injury and Risk Factors for Mortality in Patients with COVID-19 Pneumonia. Int. J. Cardiol..

[24] Paulino M.R., Moreira J.A.D.S., Correia M.G., Dos Santos L.R.A., Duarte I.P., Sabioni L.R., Mucillo F.B., Garrido R.Q., Pacheco S.L., De Lorenzo A. (2021). COVID-19 in Patients with Cardiac Disease: Impact and Variables Associated with Mortality in a Cardiology Center in Brazil. Am. Heart J. Plus Cardiol. Res. Pract..

[25] Hoffmann M., Kleine-Weber H., Schroeder S., Krüger N., Herrler T., Erichsen S., Schiergens T.S., Herrler G., Wu N.-H., Nitsche A. (2020). SARS-CoV-2 Cell Entry Depends on ACE2 and TMPRSS2 and Is Blocked by a Clinically Proven Protease Inhibitor. Cell.

[26] Verdecchia P., Cavallini C., Spanevello A., Angeli F. (2020). The Pivotal Link between ACE2 Deficiency and SARS-CoV-2 Infection. Eur. J. Intern. Med..

[27] Ferrario C.M., Jessup J., Chappell M.C., Averill D.B., Brosnihan K.B., Tallant E.A., Diz D.I., Gallagher P.E. (2005). Effect of Angiotensin-Converting Enzyme Inhibition and Angiotensin II Receptor Blockers on Cardiac Angiotensin-Converting Enzyme 2. Circulation.

[28] Lindner D., Fitzek A., Bräuninger H., Aleshcheva G., Edler C., Meissner K., Scherschel K., Kirchhof P., Escher F., Schultheiss H.-P. (2020). Association of Cardiac Infection with SARS-CoV-2 in Confirmed COVID-19 Autopsy Cases. JAMA Cardiol..

[29] Sanyaolu A., Okorie C., Marinkovic A., Prakash S., Balendra V., Lehachi A., Abbasi A.F., Haider N., Abioye A., Orish V.N. (2025). COVID-19 Management in Patients with Comorbid Conditions. World J. Virol..

[30] Abou Mansour M., El Rassi C., Sleem B., Borghol R., Arabi M. (2025). Thromboembolic Events in the Era of COVID-19: A Detailed Narrative Review. Can. J. Infect. Dis. Med. Microbiol..

[31] Ewing A.G., Salamon S., Pretorius E., Joffe D., Fox G., Bilodeau S., Bar-Yam Y. (2025). Review of Organ Damage from COVID and Long COVID: A Disease with a Spectrum of Pathology. Med. Rev..

[32] Figliozzi S., Masci P.G., Ahmadi N., Tondi L., Koutli E., Aimo A., Stamatelopoulos K., Dimopoulos M., Caforio A.L.P., Georgiopoulos G. (2020). Predictors of Adverse Prognosis in COVID-19: A Systematic Review and Meta-analysis. Eur. J. Clin. Investig..

[33] Ssentongo P., Ssentongo A.E., Heilbrunn E.S., Ba D.M., Chinchilli V.M. (2020). Association of Cardiovascular Disease and 10 Other Pre-Existing Comorbidities with COVID-19 Mortality: A Systematic Review and Meta-Analysis. PLoS ONE.

[34] Gheblawi M., Wang K., Viveiros A., Nguyen Q., Zhong J.-C., Turner A.J., Raizada M.K., Grant M.B., Oudit G.Y. (2020). Angiotensin-Converting Enzyme 2: SARS-CoV-2 Receptor and Regulator of the Renin-Angiotensin System: Celebrating the 20th Anniversary of the Discovery of ACE2. Circ. Res..

[35] Libby P., Lüscher T. (2020). COVID-19 Is, in the End, an Endothelial Disease. Eur. Heart J..

[36] Pranata R., Lim M.A., Huang I., Raharjo S.B., Lukito A.A. (2020). Hypertension Is Associated with Increased Mortality and Severity of Disease in COVID-19 Pneumonia: A Systematic Review, Meta-Analysis and Meta-Regression. J. Renin. Angiotensin. Aldosterone Syst..

[37] Lippi G., Wong J., Henry B.M. (2020). Hypertension and Its Severity or Mortality in Coronavirus Disease 2019 (COVID-19): A Pooled Analysis. Pol. Arch. Intern. Med..

[38] Ssentongo A.E., Ssentongo P., Heilbrunn E.S., Lekoubou A., Du P., Liao D., Oh J.S., Chinchilli V.M. (2020). Renin–Angiotensin–Aldosterone System Inhibitors and the Risk of Mortality in Patients with Hypertension Hospitalised for COVID-19: Systematic Review and Meta-Analysis. Open Heart.

[39] Ceriello A. (2022). Diabetes Research and Clinical Practice and the Fight against COVID-19. Diabetes Res. Clin. Pract..

[40] Huang T.-S., Chao J.-Y., Chang H.-H., Lin W.-R., Lin W.-H. (2025). COVID-19 and Diabetes: Persistent Cardiovascular and Renal Risks in the Post-Pandemic Landscape. Life.

[41] Bornstein S.R., Rubino F., Khunti K., Mingrone G., Hopkins D., Birkenfeld A.L., Boehm B., Amiel S., Holt R.I., Skyler J.S. (2020). Practical Recommendations for the Management of Diabetes in Patients with COVID-19. Lancet Diabetes Endocrinol..

[42] Apicella M., Campopiano M.C., Mantuano M., Mazoni L., Coppelli A., Del Prato S. (2020). COVID-19 in People with Diabetes: Understanding the Reasons for Worse Outcomes. Lancet Diabetes Endocrinol..

[43] Huang I., Lim M.A., Pranata R. (2020). Diabetes Mellitus Is Associated with Increased Mortality and Severity of Disease in COVID-19 Pneumonia—A Systematic Review, Meta-Analysis, and Meta-Regression. Diabetes Metab. Syndr. Clin. Res. Rev..

[44] Kumar A., Arora A., Sharma P., Anikhindi S.A., Bansal N., Singla V., Khare S., Srivastava A. (2020). Is Diabetes Mellitus Associated with Mortality and Severity of COVID-19? A Meta-Analysis. Diabetes Metab. Syndr. Clin. Res. Rev..

[45] Diao B., Wang C., Wang R., Feng Z., Zhang J., Yang H., Tan Y., Wang H., Wang C., Liu L. (2021). Human Kidney Is a Target for Novel Severe Acute Respiratory Syndrome Coronavirus 2 Infection. Nat. Commun..

[46] Ronco C., Reis T., Husain-Syed F. (2020). Management of Acute Kidney Injury in Patients with COVID-19. Lancet Respir. Med..

[47] Cheng Y., Luo R., Wang K., Zhang M., Wang Z., Dong L., Li J., Yao Y., Ge S., Xu G. (2020). Kidney Disease Is Associated with In-Hospital Death of Patients with COVID-19. Kidney Int..

[48] Vanholder R., Argiles A., Jankowski J., for the European Uremic Toxin Work Group (EUTox) (2021). A History of Uremic Toxicity and of the European Uremic Toxin Work Group (EUTox). Clin. Kidney J..

[49] Henry B.M., Lippi G. (2020). Chronic Kidney Disease Is Associated with Severe Coronavirus Disease 2019 (COVID-19) Infection. Int. Urol. Nephrol..

[50] Cai R., Zhang J., Zhu Y., Liu L., Liu Y., He Q. (2021). Mortality in Chronic Kidney Disease Patients with COVID-19: A Systematic Review and Meta-Analysis. Int. Urol. Nephrol..

[51] Cheng Y., Luo R., Wang X., Wang K., Zhang N., Zhang M., Wang Z., Dong L., Li J., Zeng R. (2020). The Incidence, Risk Factors, and Prognosis of Acute Kidney Injury in Adult Patients with Coronavirus Disease 2019. Clin. J. Am. Soc. Nephrol..

[52] Iba T., Levy J.H., Levi M., Connors J.M., Thachil J. (2020). Coagulopathy of Coronavirus Disease 2019. Crit. Care Med..

[53] Mehta P., McAuley D.F., Brown M., Sanchez E., Tattersall R.S., Manson J.J. (2020). COVID-19: Consider Cytokine Storm Syndromes and Immunosuppression. Lancet.

[54] Evans P.C., Rainger G.E., Mason J.C., Guzik T.J., Osto E., Stamataki Z., Neil D., Hoefer I.E., Fragiadaki M., Waltenberger J. (2020). Endothelial Dysfunction in COVID-19: A Position Paper of the ESC Working Group for Atherosclerosis and Vascular Biology, and the ESC Council of Basic Cardiovascular Science. Cardiovasc. Res..

[55] Gu S.X., Tyagi T., Jain K., Gu V.W., Lee S.H., Hwa J.M., Kwan J.M., Krause D.S., Lee A.I., Halene S. (2021). Thrombocytopathy and Endotheliopathy: Crucial Contributors to COVID-19 Thromboinflammation. Nat. Rev. Cardiol..

[56] Hui K.P.Y., Ho J.C.W., Cheung M., Ng K., Ching R.H.H., Lai K., Kam T.T., Gu H., Sit K.-Y., Hsin M.K.Y. (2022). SARS-CoV-2 Omicron Variant Replication in Human Bronchus and Lung Ex Vivo. Nature.

[57] Meng B., Abdullahi A., Ferreira I.A.T.M., Goonawardane N., Saito A., Kimura I., Yamasoba D., Gerber P.P., Fatihi S., Rathore S. (2022). Altered TMPRSS2 Usage by SARS-CoV-2 Omicron Impacts Infectivity and Fusogenicity. Nature.

[58] Lui K.O., Ma Z., Dimmeler S. (2024). SARS-CoV-2 Induced Vascular Endothelial Dysfunction: Direct or Indirect Effects?. Cardiovasc. Res..

[59] Ulloa A.C., Buchan S.A., Daneman N., Brown K.A. (2022). Estimates of SARS-CoV-2 Omicron Variant Severity in Ontario, Canada. JAMA.

[60] Kwan A.C., Ebinger J.E., Botting P., Navarrette J., Claggett B., Cheng S. (2023). Association of COVID-19 Vaccination with Risk for Incident Diabetes After COVID-19 Infection. JAMA Netw. Open.

[61] Kim Y.-E., Huh K., Park Y.-J., Peck K.R., Jung J. (2022). Association Between Vaccination and Acute Myocardial Infarction and Ischemic Stroke After COVID-19 Infection. JAMA.

[62] Cordero A., Santos García-Gallego C., Bertomeu-González V., Fácila L., Rodríguez-Mañero M., Escribano D., Castellano J.M., Zuazola P., Núñez J., Badimón J.J. (2020). Mortality Associated with Cardiovascular Disease in Patients with COVID-19. REC CardioClinics.

[63] Williamson E.J., Walker A.J., Bhaskaran K., Bacon S., Bates C., Morton C.E., Curtis H.J., Mehrkar A., Evans D., Inglesby P. (2020). Factors Associated with COVID-19-Related Death Using OpenSAFELY. Nature.

[64] Borges Do Nascimento I.J., Cacic N., Abdulazeem H.M., Von Groote T.C., Jayarajah U., Weerasekara I., Esfahani M.A., Civile V.T., Marusic A., Jeroncic A. (2020). Novel Coronavirus Infection (COVID-19) in Humans: A Scoping Review and Meta-Analysis. J. Clin. Med..

[65] Page M.J., McKenzie J.E., Bossuyt P.M., Boutron I., Hoffmann T.C., Mulrow C.D., Shamseer L., Tetzlaff J.M., Akl E.A., Brennan S.E. (2021). The PRISMA 2020 statement: An updated guideline for reporting systematic reviews. BMJ.

